# The Assessment of Cardiovascular Abnormalities in Patients With Chronic Liver Disease: A Cross-Sectional Study

**DOI:** 10.7759/cureus.73311

**Published:** 2024-11-09

**Authors:** Kunjan PareshKumar Shah, Sailaja Kuruvada, Mridulkrishnan Gopalakrishnan, Bhargavi Latchireddy, Talla Prathima, Niyati Patra

**Affiliations:** 1 Department of General Medicine, Dr. N.D. Desai Faculty of Medical Science and Research, Nadiad, IND; 2 Department of General Medicine, University College London, London, GBR; 3 Department of General Medicine, Kasturba Medical College, Manipal, Manipal, IND; 4 Department of Gastroenterology, Craigavon Area Hospital, Craigavon, GBR; 5 Department of General Medicine, Hebei Medical University, Shijiazhuang, CHN; 6 Department of Emergency Medicine, Caboolture Hospital, Caboolture, AUS

**Keywords:** cardiac abnormalities, child-turcotte-pugh scores, chronic liver disease, left ventricular diastolic dysfunction, meld scores, qtc prolongation

## Abstract

Background

Chronic liver disease (CLD) is associated with a wide range of systemic complications, including cardiovascular abnormalities. This study aimed to assess the prevalence of cardiac abnormalities and to correlate with the severity of liver disorder.

Materials and methods

A cross-sectional analysis comprising 120 adult subjects diagnosed with CLD was performed. Data were collected through clinical assessments, including liver function tests, echocardiography, and electrocardiograms (ECG). The severity of CLD was determined using the Child-Turcotte-Pugh (CTP) and Model for End-Stage Liver Disease (MELD) scoring systems.

Results

In this study, 85 (70.8%) were men, and the major cause of CLD was alcoholism in 89 (66.7%) of the patients. Regarding CLD severity, the majority of patients were in MELD stage 2, and 70 (58.3%) and 76 (63.3%) were in CTP class C. The prevalence of various cardiac abnormalities was as follows: left ventricular systolic dysfunction (LVSD) in 25 (20.8%), left ventricular diastolic dysfunction (LVDD) in 76 (63.3%), and prolonged corrected QT (QTc) in 52 (43.3%) of the CLD patients. The values of the QTc interval were higher in MELD stage 3, and it was significant (p=0.001). The association between LVSD (p=0.004) and LVDD (p=0.001) was significant, and the proportion of CLD subjects with cardiac dysfunction was greater in CTP class C compared to classes B and A.

Conclusion

The present study highlights a concerning prevalence of cardiovascular abnormalities among chronic liver disease patients, underscoring the need for routine cardiovascular assessment in this population.

## Introduction

Chronic liver disease (CLD) constitutes a major health issue, impacting millions of individuals globally. The condition is marked by the progressive degeneration of hepatic tissue, resulting in compromised liver functionality and the potential emergence of severe, life-threatening complications. The prevalence of CLD has been steadily increasing in recent years, with risk factors including excessive alcohol consumption, viral hepatitis, obesity, and certain medications. The CLD has a marked impact on patients' quality of life and affects their overall health, making it crucial to understand its causes, symptoms, and treatment options [1]. The etiology of liver disease is multifactorial; however, the four principal contributors to the burden associated with CLD are chronic hepatitis C (CHC) virus, chronic hepatitis B (CHB) virus, alcoholic liver disease (ALD), and nonalcoholic fatty liver disorder (NAFLD) [2]. The recent outcomes from the Global Burden of Disease study conducted in 2019 indicate that CLD constitutes 1.8% of the global disease burden. Furthermore, it was observed that CLD has contributed to a 33% increase in disability-adjusted life years [3]. A recent systematic review conducted in India reveals that the major contributors to CLD are alcohol with an estimate of 43.2%, NAFLD at 14.4%, hepatitis B virus at 11.5%, and hepatitis C virus (HCV) at 6.2% [4].

Recent investigations have revealed a notable association between cardiovascular disease (CVD) and chronic liver disease (CLD) [5]. Cardiac dysfunction associated with liver disease was originally ascribed to alcohol-induced liver pathology. Current understanding indicates that this phenomenon is observed throughout the entire spectrum of liver disease, regardless of the underlying etiology of liver dysfunction, and is referred to as cirrhotic cardiomyopathy (CCM) [6]. The hyperdynamic state in cirrhosis subjects is represented by elevated cardiac output and plasma volume and a reduction in systemic vascular resistance and blood pressure [7]. First, the diminished left ventricular efficiency observed in patients with cirrhosis was attributed to the direct toxic effects of alcohol [8]. Research on ventricular diastolic filling in the context of cirrhosis indicates the existence of a subclinical CVD characterized by diastolic dysfunction and a reduced E/A ratio [9]. Additionally, there was evidence of diminished cardiac contractility characterized by systolic and diastolic dysfunction and electromechanical defects, including a prolonged QT interval in patients with CLD [10]. In this backdrop, the present study was conducted to evaluate cardiac abnormalities in patients with chronic liver disease and correlate with the disease severity.

## Materials and methods

A cross-sectional study was performed among 120 participants diagnosed with chronic liver disease at the Department of General Medicine, Dr. N.D. Desai Faculty of Medical Science and Research, India. The study was conducted for a period of one year, from August 2023 to 2024. The study was approved by the Institutional Ethical Clearance Committee of Dr. N.D. Desai Faculty of Medical Science and Research with protocol number NDDFMSR/IEC/285/2023.

Inclusion criteria

Patients aged more than 18 years with the presence of CLD of different pathologies, an ultrasound examination of the liver with the incidence of nodules showing clinical manifestations of portal hypertension, and a biopsy examination showing the cirrhosis subjects were included in this study.

Exclusion criteria

Subjects with a previous medical history indicative of cardiovascular conditions were excluded from the study. Individuals with comorbid conditions that may impact cardiac function, such as diabetes and hypertension, were also excluded from the study.

The categorization of chronic liver disease (CLD) was determined through a thorough evaluation encompassing clinical history, physical examination, biochemical tests including liver function tests, and serological assessments (hepatitis B surface antigen (HBsAg) and HCV), in addition to ultrasonography imaging methodologies. For each selected case, a thorough medical history was collected, accompanied by a detailed general and systemic examination. Following this, all patients received an electrocardiogram (ECG) and 2D echocardiography assessment to evaluate cardiac function and condition. The CLD patients were subjected to ECG analysis, and the corrected QT (QTc) values were measured using the following formula: QTc=QT (seconds)/√RR (seconds). The QTc was prolonged when the value was >0.44 seconds (440 ms).

Systolic dysfunction

Ejection fraction (EF) was evaluated by the Simpson formula: LVEF (%)=(EDV-ESV/EDV)×100, where end-diastolic volume (EDV) is the volume of blood in the left ventricle at the end of filling (diastole), end-systolic volume (ESV) is the volume of blood remaining in the left ventricle after contraction (systole), and left ventricular ejection fraction (LVEF) of less than 55% suggests an impaired systolic function.

Diastolic dysfunction

Diastolic dysfunction was evaluated by measuring the E/e' and E/A ratio, isovolumic relaxation time (IVRT), E-wave deceleration time (EDT), and pulmonary flow measurements. No individual variable is unequivocally superior to others. A combination approach was employed, utilizing the parameters E/e' and E/A in the present study.

Four-chamber views were obtained and optimized. The assessment of left ventricular diastolic performance was conducted through Doppler examination, focusing on the trans-mitral velocities during early (E) and late (A) left ventricular filling phases. Utilizing the mitral inflow signal, we quantified the E velocity (E) and the A velocity (A) and calculated the E/A ratio pertaining to early diastolic left ventricular filling. Pulsed wave tissue Doppler imaging (TDI) was conducted at the interface of the septal mitral annulus. Early diastolic velocities (e' medial) were measured, and the E/e' medial ratio was computed. Patients were categorized according to the presence or absence of left ventricular diastolic dysfunction, following the criteria set forth by the American Society of Echocardiography (ASE). Left ventricular diastolic dysfunction (LVDD) is considered by an E/A ratio ranging from 0.8 to 1.5 cm/second, accompanied by a septal e' measurement of less than 8 cm/second. The absence of the condition was indicated by an E/A ratio exceeding 1 and a septal e' velocity greater than 8 cm/second.

The diastolic dysfunction was assessed as follows: grade 1, impaired relaxation (mild diastolic dysfunction); grade 2, pseudonormalization (moderate diastolic dysfunction); and grade 3, restrictive filling (severe diastolic dysfunction).

Further, the patients underwent evaluation utilizing the Child-Turcotte-Pugh (CTP) and Model for End-Stage Liver Disease (MELD) scoring system: MELD=3.78×ln (bilirubin)+11.2×ln (creatinine)+9.57×ln (INR)+6.43

MELD scoring was as follows: stage 1 MELD score, <9; stage 2 MELD score, 10-19; and stage III MELD score, >20 [11]. The CTP scores were measured using an online calculator. The CTP score comprised two continuous variables (bilirubin and albumin) and three discrete variables (ascites, encephalopathy, and international normalized ratio {INR}): scores 5-6, class A (well-compensated disease); scores 7-9, class B (significant liver disease); and scores 10-15, class C (decompensated liver disease) [12].

Statistical analysis

The continuous data and the categorical data were represented by mean (SD) and frequency (%). Pearson's chi-square test was used to evaluate the association between the categorical variables. A one-way ANOVA was utilized when more than two continuous variables were available. A p value of <0.05 was considered statistically significant. The analysis was conducted using the SPSS statistical analysis software version 27 (IBM Corp., Armonk, NY).

## Results

The mean age of the CLD subjects was 56.34±11.76 years, and the majority of the subjects (75, 62.5%) were in the age group 40-60 years. Male preponderance was observed, which accounts for 85 (70.8%), and the mean BMI was 27.81±4.28 kg/m^2^. The most common etiology for the CLD was alcoholism in 80 (66.7%) of the subjects. The mean respiratory rate between the CLD subjects was 15.45±2.87 breaths/minute. The aspartate aminotransferase (AST) and alanine aminotransferase (ALT) levels were 155.46±7.38 IU/L and 164.12±8.45 IU/L, respectively, and the total bilirubin level was 7.12±1.28 mg/dL. The mean sodium level was 136.28±9.36 mEq/L, and the potassium level was 4.46±0.76 mEq/L. The mean MELD score was 14.27±5.20, and the majority of the patients (70, 58.3%) were in MELD stage 2. The mean CTP score was 8.18±1.87, and the majority of the patients (76, 63.3%) had CTP class C. The demographic characteristics of the CLD subjects are shown in Table 1.

**Table 1:** Demographics and clinical characteristics of the CLD subjects Data are shown as mean±SD, frequency (n), and percentage (%) CTP, Child-Turcotte-Pugh; MELD, Model for End-Stage Liver Disease; SpO_2_, oxygen saturation; BP, blood pressure; AST, aspartate aminotransferase; ALT, alanine aminotransferase; INR, international normalized ratio; CLD, chronic liver disease

Parameters	Values
Age in years (mean±SD)	56.34±11.76
Gender (n, %)	
Male	85 (70.8%)
Female	35 (29.2%)
Etiology	
Alcoholism	80 (66.7%)
Hepatitis B virus	21 (17.5%)
Hepatitis C virus	16 (13.3%)
Hepatic carcinoma	3 (2.5%)
Vitals	
Respiratory rate (breaths/minute) (mean±SD)	15.45±2.87
Pulse (beats/minute) (mean±SD)	85.12±12.96
SpO_2_ (%)	98.50±1.31
Systolic BP (mm/Hg) (mean±SD)	125.72±12.84
Diastolic BP (mm/Hg) (mean±SD)	78.98±7.12
Hepatic markers	
AST (IU/L) ( mean±SD)	155.46±7.38
ALT (IU/L) ( mean±SD)	164.12±8.45
Total bilirubin (mg/dL) (mean±SD)	7.12±1.28
Albumin (g/dL) (mean±SD)	2.78±0.59
INR (mean±SD)	1.25±0.12
Renal markers	
Sodium (mEq/L) (mean±SD)	135.28±9.36
Potassium (mEq/L) (mean±SD)	4.46±0.76
MELD score (mean±SD)	14.27±5.20
MELD stage (n, %)	
Stage 1	35 (29.2%)
Stage 2	70 (58.3%)
Stage 3	15 (12.5%)
CTP score (mean±SD)	8.18±1.87
CTP score category (n, %)	
CTP class A	9 (7.5%)
CTP class B	35 (29.2%)
CTP class C	76 (63.3%)

The mean left ventricular ejection fraction was 56.42%±7.39%. The E/A ratio and E/e' ratio were 0.97±0.27 and 13.5±2.87, respectively. The mean left ventricular systolic diameter was 3.10±0.56 cm, and the left ventricular diastolic diameter was 4.82±0.72 cm. The mean QTc interval was 472.28±65.18 ms. The cardiac parameters among the CLD patients are shown in Table 2.

**Table 2:** Measurement of cardiac parameters in CLD patients Data are shown as mean±SD QTc, corrected QT; CLD, chronic liver disease

Parameters	Values
Left ventricular ejection fraction, % (mean±SD)	56.42±7.39
E/A ratio (mean±SD)	0.97±0.27
E/e' ratio (mean±SD)	13.5±2.87
Left ventricular systolic diameter, cm (mean±SD)	3.10±0.56
Left ventricular diastolic diameter, cm (mean±SD)	4.82±0.72
QT interval, ms (mean±SD)	385.92±56.31
QTc, ms (mean±SD)	472.28±65.18

In this study, out of 120 CLD patients, left ventricular systolic dysfunction (LVSD) was present in 25 (20.8%), left ventricular diastolic dysfunction (LVDD) in 76 (63.3%), and prolonged QTc in 52 (43.3%) of the patients (Table 3).

**Table 3:** Distribution of cardiac abnormalities in CLD patients Data are shown as frequency (%) QTc, corrected QT; CLD, chronic liver disease

Cardiac abnormalities	Frequency (%)
Left ventricular systolic dysfunction (n, %)	
Present	25 (20.8%)
Absent	95 (79.2%)
Left ventricular diastolic dysfunction (n, %)	
Present	76 (63.3%)
Absent	44 (36.7%)
QTc prolongation	
Present	52 (43.3%)
Absent	68 (56.7%)

In the present study, the QTc interval was greater in MELD stage 3 than in MELD stage 2 and MELD stage 1 (468.31±34.87 ms versus 442.57±29.34 ms and 403.65±20.42 ms), and it was significant (p=0.001) (Table 4).

**Table 4:** Comparison of QTc interval in MELD stages among the CLD patients Data are shown as mean±SD *Significant, p<0.05, one-way ANOVA MELD, Model for End-Stage Liver Disease; QTc, corrected QT; CLD, chronic liver disease

MELD stage (n, %)	QTc interval (ms)	F value	P value
Stage 1 (n=35)	403.65±20.42	54.12	0.001*
Stage 2 (n=70)	442.57±29.34		
Stage 3 (n=15)	468.31±34.87		

The association between left ventricular systolic dysfunction (LVSD) and CTP scores was also studied. In this study, out of 25 CLD patients with LVSD, the majority of them (14, 56%) were in the CTP class C category, eight (32%) in the class B category, and three (12%) in the class A category. Overall, this association was found to be significant (p=0.004). The results are shown in Table 5.

**Table 5:** Association between left ventricular systolic dysfunction (LVSD) and CTP Data are shown as frequency (%) *Significant, p<0.05 (chi-square test)

LVSD	Child-Turcotte-Pugh (CTP) scores			Total	Chi-square (χ^2^) and P value
	Class A (n=9)	Class B (n=35)	Class C (n=76)		
Present	3 (12%)	8 (32%)	14 (56%)	25 (100%)	χ^2^=24.65; p=0.004*
Absent	6 (6.3%)	27 (28.4%)	62 (65.3%)	95 (100%)	

The association between left ventricular diastolic dysfunction (LVDD) and CTP scores was also studied. In this study, out of 76 CLD patients with LVDD, the majority of them (52, 68.4%) were in the CTP class C category, 18 (23.7%) in the class B category, and six (7.9%) in the class A category. Overall, this association was found to be significant (p=0.001) (Table 6).

**Table 6:** Association between left ventricular diastolic dysfunction (LVDD) and CTP Data are shown as frequency (%) *Significant, p<0.05 (chi-square test)

LVDD	Child-Turcotte-Pugh (CTP) scores			Total	Chi-square (χ^2^) and P value
	Class A (n=9)	Class B (n=35)	Class C (n=76)		
Present	6 (7.9%)	18 (23.7%)	52 (68.4%)	76 (100%)	χ^2^=32.12; p=0.001*
Absent	3 (6.8%)	17 (38.6%)	24 (54.6%)	44 (100%)	

**Figure 1:**
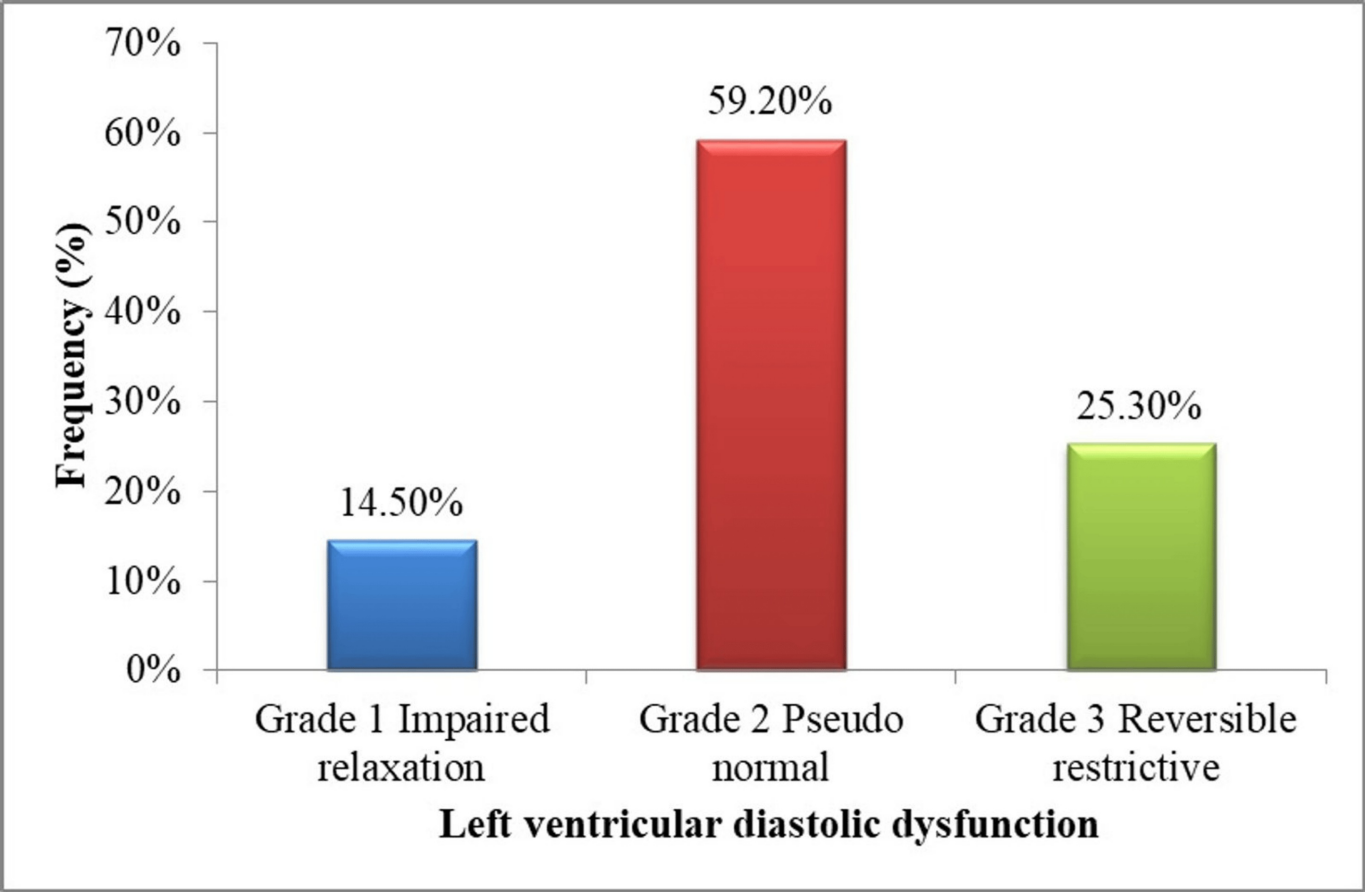
Distribution of CLD patients according to left ventricular diastolic dysfunction grades CLD: chronic liver disease

In our study, out of 76 CLD patients with left ventricular diastolic dysfunction (LVDD), 11 (14.5%) patients were in grade 1, impaired relaxation; 45 (59.2%) were in grade 2, pseudonormal; and 20 (25.3%) were in grade 3, reversible restrictive (Figure 1).

## Discussion

Cardiovascular abnormalities are relatively common in subjects with chronic liver disease (CLD), especially as the disease progresses to more severe stages. The liver and heart have a close physiological relationship, so when the liver is diseased, it often affects the cardiovascular system through various mechanisms, including systemic inflammation, hormonal imbalances, and blood flow changes [13]. The various cardiac abnormalities in CLD patients are QTc interval prolongation, and it is closely associated with the intensity of cirrhosis [14]. Echocardiographically, left ventricular diastolic dysfunction is the primary cardiac alteration identified in individuals with cirrhosis and serves as a main diagnostic criterion for cirrhotic cardiomyopathy [15]. Disruptions in systolic function are more challenging to identify, as they are mild at rest and may only become apparent under physiological stress or pharmacological intervention [16]. So, this study was carried out to evaluate the cardiac abnormalities in CLD subjects with different etiologies.

The current study revealed the mean age of the patients at 56.34±11.76 years, with a notable male predominance comprising 85 (70.8%) of the total population analyzed. Likewise, in the research done by Yousaf et al., the mean age of the cirrhotic subjects with cardiac abnormalities was 57.14±9.84 years [17]. In a study conducted by Sharma and Kavya, the majority of the CLD patients with cardiac manifestations were men, which constitute 83% of the total study sample [18].

In our study, the mean MELD score was 14.27±5.20, and most of the subjects (70, 58.3%) were in MELD stage 2. In the current study, the mean CTP score was 8.18±1.87, and the majority of the patients (76, 63.3%) had a CTP score in class C. Similarly, in a study done by Sharma and Kavya, the majority of CLD patients (71%) were in CTP class C [18]. In the present study, the major cause of CLD was chronic alcoholism in 66.7% of the cases. Likewise, in the study by Sharma and Kavya, the major etiological factor for CLD was alcoholism in 80% of the subjects [18].

In the current study, the mean LVEF was 56.42%±7.39%, and the E/A ratio and E/e' ratio were 0.97±0.27 and 13.5±2.87, respectively. This indicates the proportion of blood ejected from the left ventricle with each heartbeat. Generally, an LVEF of 55%-70% is considered normal, so a mean of 59.42% falls within the normal range in the present study. The E/A ratio reflects the diastolic function of the heart, specifically the filling of the left ventricle during early (E) and late (A) diastole. An E/A ratio of around 1 is often seen as borderline and may suggest early signs of diastolic dysfunction in CLD patients. The E/e' ratio is used to assess left ventricular filling pressure, with higher values potentially indicating increased filling pressures and possibly diastolic dysfunction. An E/e' ratio of above 15 suggests elevated filling pressures, while values between 8 and 15 are more intermediate. In this present study, the mean value of 13.5 is relatively high and may suggest increased filling pressures in CLD patients [19]. Likewise, in a study done by Solanki et al., the mean LVEF (%) was 64.6±7.8, the E/A ratio was 9.1±2.1, and the E/e' ratio was 9.3±3.0 [19]. In this current study, the mean QTc interval in CLD patients was 472.28±65.18 ms, and it indicates prolonged repolarization, which is commonly observed due to associated electrolyte imbalances, autonomic dysfunction, and potential cirrhotic cardiomyopathy. Prolonged QTc is significant as it can increase the risk of arrhythmias, specifically torsades de pointes, which may lead to sudden cardiac events. Monitoring and managing QTc prolongation are essential for mitigating cardiovascular risks in CLD patients, particularly in advanced stages or during medical treatments that may influence QT intervals [20].

In the present study, in CLD patients, the prevalence of left ventricular systolic dysfunction (LVSD) was 25 (20.8%), left ventricular diastolic dysfunction (LVDD) 76 (63.3%), and prolonged QTc 52 (43.3%). In a study done by Sharma and Kavya, the prevalence of LVSD and LVDD in liver cirrhosis patients was 8% and 43% [18]. In another study done by Yousaf et al., the prevalence of LVDD was 71.8%, and QTc prolongation was 78% [17]. The study by Zhang et al. showed the prevalence of LVDD of up to 55%-70% in advanced CLD patients, with LVSD being rarer, often under 30%. Thus, it delineates that diastolic dysfunction is a common feature of cirrhotic cardiomyopathy, likely tied to structural and functional heart changes driven by liver disease progression [21].

In studies on cardiac abnormalities in CLD patients, the prevalence of prolonged QTc intervals varies. One study reported a prevalence of 48.9% in cirrhotic patients [22]. Another study found that QTc prolongation was observed in 40%-60% of patients with advanced liver disease, indicating a consistent concern regarding cardiac function in this population​ [23]. These outcomes highlight the significance of cardiac monitoring in CLD subjects to mitigate risks associated with prolonged QTc intervals.

In the present study, the QTc interval was higher in MELD stage 3 as in stages 2 and 1, and it was significant (p=0.001). The QTc interval tends to increase with the severity of liver disease often evaluated using the MELD score. Studies show that patients with greater MELD scores, demonstrating more severe liver disorder, generally have longer QTc intervals. This correlation suggests that as liver disease progresses, the QTc prolongation also increases and leads to the development of cardiac arrhythmias. Similar to our report, Patil et al. stated that there was a substantial increase in the QTc prolongation in MELD stage 3 (458.53 ms) when compared to stage 2 (439.03) and stage 3 (407.38±21.65), and it was significant (p<0.0001) [24]. In a recent research performed by Kumar et al., a significant correlation between QTc interval and MELD in cirrhosis subjects was documented [25].

In our study, there was a significant correlation between left ventricular systolic dysfunction (LVSD) and CTP scores in CLD patients (p=0.004). The frequency of LVSD was higher with patients in CTP class C as compared to CTP class B and class A (14 {56%} versus eight {32%} and three {12%}). Studies have shown that CLD patients with CTP scores in classes B and C (44% and 33.4%) have a significantly higher prevalence of LVSD than those with CTP class A (22.2%) [26].

A significant correlation between left ventricular diastolic dysfunction (LVDD) and CTP scores in CLD subjects (p=0.001) was observed in this study. The frequency of LVSD was higher with patients in CTP class C as compared to CTP class B and class A (52 {68.4%} versus 18 {23.7%} and six {7.9%}). In patients with high CTP scores, which indicate more severe liver disease, LVDD prevalence tends to be greater due to factors such as increased cardiac afterload, altered myocardial metabolism, and autonomic dysfunction [27]. Solanki et al. documented that CLD subjects with higher CTP classes are more likely to have LVDD, 79.2% of CTP class C compared to CTP class B and CTP class A (65.2% and 35%, respectively), and it was significant (p=0.001) [19]. In this study, CLD patients with LVDD (45, 59.2%) were in grade 2, pseudonormal. Similarly, in a study done by Angadi et al., the majority of the CLD patients (64.28%) were in grade 2, pseudonormal LVDD [28].

Limitations

The study is characterized by a relatively small sample size and is conducted at a single center. Being cross-sectional, the study may struggle to establish causality between CLD and cardiovascular abnormalities. In addition, the association between inflammatory markers and cardiac abnormalities was not measured.

## Conclusions

In conclusion, our study highlights the significant impact of chronic liver disease on cardiovascular health and the importance of early intervention in improving outcomes for these patients. This study demonstrated a greater prevalence of left ventricular diastolic dysfunction in comparison to left ventricular systolic dysfunction. Further, the prolongation of QTc was also observed, which is suggestive of arrhythmic conditions.
